# What is the impact of child abuse on gray matter abnormalities in individuals with major depressive disorder: a case control study

**DOI:** 10.1186/s12888-016-1116-y

**Published:** 2016-11-14

**Authors:** Sung Jun Ahn, Sunghyon Kyeong, Sang Hyun Suh, Jae-Jin Kim, Tae-Sub Chung, Jeong-Ho Seok

**Affiliations:** 1 Department of Radiology, Gangnam Severance Hospital, Yonsei University College of Medicine, 211 Eonju-ro, Gangnam-gu, Seoul 06273 Republic of Korea; 2 Department of Psychiatry, Gangnam Severance Hospital, Institute of Behavioral Science in Medicine, Yonsei University College of Medicine, 211 Eonju-ro, Gangnam-gu, Seoul 06273 Republic of Korea

**Keywords:** Major depressive disorder, Physical abuse, Emotional abuse, Voxel-based morphometry, Orbitofrontal cortex, Emotional dysregulation

## Abstract

**Background:**

Patients with major depressive disorder (MDD) present heterogeneous clinical symptoms, and childhood abuse is associated with deepening of psychopathology. The aim of this study was to identify structural brain abnormalities in MDD and to assess further differences in gray matter density (GMD) associated with childhood abuse in MDD.

**Methods:**

Differences in regional GMD between 34 MDD patients and 26 healthy controls were assessed using magnetic resonance imaging and optimized voxel-based morphometry. Within the MDD group, further comparisons were performed focusing on the experience of maltreatment during childhood (23 MDD with child abuse vs 11 MDD without child abuse).

**Results:**

Compared with healthy controls, the MDD patient group showed decreased GMD in the bilateral orbitofrontal cortices, right superior frontal gyrus, right posterior cingulate gyrus, bilateral middle occipital gyri, and left cuneus. In addition, the patient group showed increased GMD in bilateral postcentral gyri, parieto-occipital cortices, putamina, thalami, and hippocampi, and left cerebellar declive and tuber of vermis. Within the MDD patient group, the subgroup with abuse showed a tendency of decreased GMD in right orbitofrontal cortex, but showed increased GMD in the left postcentral gyrus compared to the subgroup without abuse.

**Conclusions:**

Our findings suggest a complicated dysfunction of networks between cortical-subcortical circuits in MDD. In addition, increased GMD in postcentral gyrus and a possible reduction of GMD in the orbitofrontal cortex of MDD patients with abuse subgroup may be associated with abnormalities of body perception and emotional dysregulation.

**Electronic supplementary material:**

The online version of this article (doi:10.1186/s12888-016-1116-y) contains supplementary material, which is available to authorized users.

## Background

Although major depressive disorder (MDD) is one of the leading causes of disease burden all around the world, the etiology and pathophysiology remain uncertain [1]. Advanced magnetic resonance imaging (MRI) has been used to identify the key brain regions involved in MDD. Based on this work, several brain networks were suggested to be associated with pathophysiology of MDD. First, dysfunction in limbic-cortical circuitry, including the orbitofrontal cortex (OFC), dorsolateral prefrontal cortex (DLPFC), insular and temporal pole may be associated with MDD [2–4]. Second, network dysfunction between dorsal (dorsomedial prefrontal cortex, DLPFC, dorsal anterior cingulate cortex (ACC), posterior cingulate cortex), and ventral (subgenual anterior cingulate, amygdala) components, as well as the rostral ACC, which connects the two components, is suggested in the pathophysiology of MDD [5, 6]. Third, cortico-subcortical circuitry including the limbic-cortical-striatal-pallidal-thalamic circuit and also the medial and orbitofrontal cortex and their extended cortical circuits, may be associated with development of MDD [2, 7]. In contrast to the large number of imaging studies supporting the first two networks, there has been less evidence supporting the cortico-subcortical circuitry.

People who have experienced child abuse or maltreatment have an increased risk of MDD, and the impact of these experiences may extend throughout a person’s lifetime [8–11]. The emotional dysregulation may be a central risk pathway to MDD following child abuse [12]. Bradley et al., showed that child abuse altered the endogenous stress response, principally corticotropin-releasing hormone [13]. Child abuse exhibited increased amygdala connectivity with hippocampus and prefrontal cortex, implying alteration to frontolimbic circuitry, one of circuitry in MDD [14]. However, it is still uncertain how child abuse affects brain networks in the pathophysiology of MDD.

Voxel-based morphometry (VBM) has become an established research method with which to explore brain structure differences, and to elucidate brain networks involved in specific diseases [15]. Since previous neuroimaging studies investigating the pathophysiology of MDD have reported network dysfunction between cortical and subcortical structures [2, 7], we predicted that gray matter abnormalities in cortico-subcortical circuitry would be observed in individuals with MDD in this study. In addition, we assessed the GM density (GMD) differences depending on presence or absence of childhood abuse within MDD subjects.

## Methods

### Subjects

Thirty-four patients with MDD and 26 healthy controls were recruited from the Department of Psychiatry outpatient clinic at Gangnam Severance Hospital. The two groups were matched by age, gender, and education. All participants were right-handed. Depending on exposure to physical or emotional abuse in childhood, patients with MDD were divided into two subsets (23 MDD patients with child abuse and 11 MDD patients without child abuse).

All patients were interviewed with the Structured Clinical Interview for DSM-IV. They met the MDD in DSM-IV criteria. Patients were excluded if they had a history of seizures, head trauma, dementia, intellectual impairment, neurological disease or neurosurgery, substance abuse or dependence, chronic medical conditions, history of learning disabilities, psychotic symptoms, or cardiovascular disease. Healthy control subjects had no personal history of psychiatric illness and also met the exclusion criteria outlined above.

The study was approved by the institutional review board (IRB) of Gangnam Severance Hospital, and all participants gave written informed consent after a complete description of the study was provided to them.

### Assessment

Depressive symptom severity of the participants was assessed using the Korean version of the Beck Depression Inventory (BDI) [16]. Screening for child abuse was conducted with a “yes” or “no” questionnaire asking about the presence of traumatic physical or emotional abuse experience during childhood. Patients reporting “yes” answers to both physical and emotional abuse experience were included into abuse subgroup and patients reporting “no” answers to both abuse screening questionnaire into non-abuse subgroup. Patients reporting equivocal answers to abuse screening questionnaire were excluded for further evaluation. Detailed inquiry about child abuse or stressful experiences in early-life was conducted using the Korean Childhood Abuse Experience Questionnaire developed by Oh [17]. This questionnaire has 44 items that inquire about exposure to five categories of stressful early-life experiences, including emotional abuse (five items), physical abuse (nine items), neglect (ten items), and inter-parental violence (ten items) from the Parent-child Conflict Tactics Scale [18], and ten items regarding to sexual abuse from another scale developed by Korean researchers [19].

### MRI protocol

Patients were imaged with a 3.0T MR unit (Discovery MR750; GE Healthcare, Milwaukee, WI). Three-dimensional Spoiled Gradient Echo (3D-SPGR) sequences (TR/TE = 8.29/3.28; thickness = 1, field of view = 220 × 220, matrix = 256 × 256 [reconstructed to 512 × 512], flip angle = 12, reconstructed voxel = 0.430 × 0.430 × 1 mm) were used for structural image.

### Image analysis

All the structural MRI post-processing was performed by a single experienced observer, unaware of patients’ information. Regional volumetric measurements were conducted on the 3D-SPGR images, using an optimized VBM protocol and the statistical parametric mapping (SPM 12) software [20].

The 3D MR datasets of all patients and controls were processed using following steps: (1) custom template creation: a study-specific whole brain T1-weighted template and prior images of gray matter (GM), white matter (WM), and cerebrospinal fluid (CSF) were created based on the Montreal Neurological Institute (MNI) template in SPM12. (2) Segmentation: each participant’s original image was spatially normalized based on the customized template and subsequently segmented into GM, WM, and CSF based on the customized priors. (3) Smoothing: the normalized and segmented GM images were re-sliced by 1.5 mm × 1.5 mm × 1.5 mm voxels and smoothed using a Gaussian kernel of 8 mm full-width at half-maximum.

### Statistical analysis

A two-sample *t*-test was performed using SPM12 to compare the difference of GMD between MDD patients and healthy controls while controlling for age, gender, and total gray matter volume (TGMV) effects. Statistical significances for group differences were set at threshold of AlphaSim corrected *p* < 0.05, which corresponds to an uncorrected *p* < 0.001 and cluster size k >128 voxels. The extent of cluster size was estimated through a MonteCarlo simulation using AFNI’s AlphaSim program [21] with 10,000 iterations. Additionally, within the MDD group, further comparison focusing on the experience of childhood abuse was conducted using SPSS 20.0 software (IBM Corp. Released 2011. IBM SPSS Statistics for Windows, Version 20.0. Armonk, NY: IBM Corp.). The confounding effects from age, gender, TGMV, and BDI score were controlled in comparisons of MDD patients with and without childhood abuse. A *p*-value less than 0.05 was considered statistically significant.

## Results

### Clinical characteristics

There were no significant differences in demographic characteristics such as age, gender, education year between the subjects in this study. Depression severity score was significantly higher in the MDD patient group than in the healthy control group. As shown in Table 1, MDD patients with abuse subgroup reported more stressful experiences in their early life compared to individuals in the other two groups (i.e., MDD without abuse or healthy controls).

**Table 1:** Clinical characteristics of the participants

	MDD		CONT		Post hoc**	
	MDD with abuse^a^ (*N* = 23)	MDD without abuse^b^ (*N* = 11)	Healthy controls^c^ (*N* = 26)	p*	Comparison	(p)
Male/Female	3/20	2/9	7/19	0.484		
Age (years)	32.3 ± 7.4	32.7 ± 8.5	31.4 ± 7.6	0.866		
Education (years)	14.8 ± 2.1	16.0 ± 2.3	16.0 ± 1.6	0.090		
Beck depression inventory score	32.3 ± 11.8	27.6 ± 5.2	3.0 ± 3.4	<0.001	a>c	<0.001
					b>c	<0.001
Early-life stress factor score						
Emotional abuse	12.3 ± 4.6	1.7 ± 1.6	1.9 ± 2.0	<0.001	a>b	<0.001
					a>c	<0.001
Physical abuse	7.4 ± 5.6	0.9 ± 0.9	1.3 ± 0.9	<0.001	a>b	<0.001
					a>c	<0.001
Sexual abuse	0.7 ± 1.0	0.2 ± 0.4	0.05 ± 0.1	0.003	a>c	0.002
Neglect	3.1 ± 3.9	0.2 ± 0.5	0.2 ± 0.6	<0.001	a>b	0.008
					a>c	<0.001
Domestic violence	1.6 ± 1.2	0.9 ± 0.6	0.2 ± 0.3	<0.001	a>c b>c	<0.001 0.038

*Note*: *CONT* control group, *MDD* major depressive disorder patient group

*Fisher’s exact test for gender variable was done; Analyses of variance test for continuous variable were done

**post-hoc test with Bonferroni’s method (^a^: MDD with abuse, ^b^: MDD without abuse, ^c^: healthy controls)

*p*-value less than 0.05 was considered significant

### Voxel-based morphometry

**Fig. 1:**
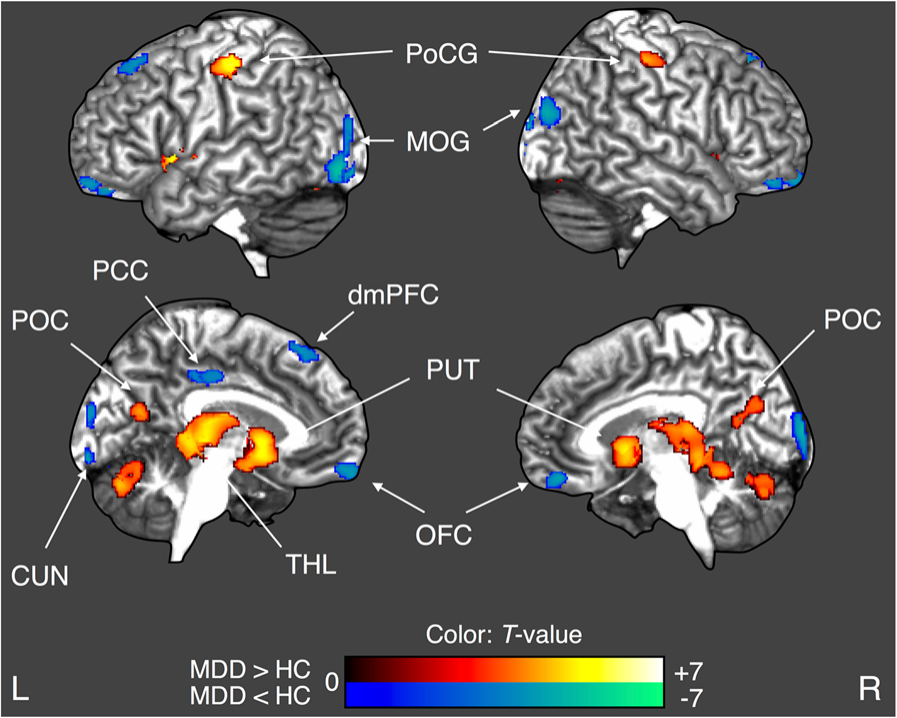
Statistical comparisons of GMD between MDD patients and healthy controls (HC). Clusters colored with red (*blue*) indicate the increased (decreased) GMD in MDD patients. Abbreviations: GMD, gray matter density; MDD, major depressive disorder; HC, healthy controls; CUN, cuneus; dmPFC, dorsomedial prefrontal cortex; L, left; MOG, middle occipital gyrus; OFC, orbitofrontal cortex; PCC, posterior cingulate cortex; POC, parieto-occipital cortex; PoCG, postcentral gyrus; PUT, putamen; R, right; THL, thalamus

Compared with healthy controls, the MDD patient group showed decreased GMD in the bilateral orbitofrontal cortices, right superior frontal gyrus, right posterior cingulate gyrus, bilateral middle occipital gyri, and left cuneus. This group also showed increased GMD in the bilateral postcentral gyri, parieto-occipital cortices, putamina, thalami, and hippocampi, and left cerebellar declive and tuber of vermis (Table 2 and Fig. 1).

**Table 2:** Regions of significantly different GMD in MDD patients compared to healthy controls

Anatomical Region	Side	BA	MNI coordinate, mm			Number of voxel	Zscore
			x	y	z		
Increased gray matter density in MDD							
Postcentral gyrus	L	4	−39	−27	52	210	3.99
Postcentral gyrus	R	4	36	−24	54	518	5.05
Parieto-occipital cortex	L	31	−8	−70	26	140	3.45
Parieto-occipital cortex	R	23	4	−64	18	166	3.91
Putamen	L		−26	4	−4	1219	4.90
Putamen	R		26	8	−6	2174	5.05
Thalamus	R		4	−27	2	3687	4.80
Hippocampus	L		−16	−36	−4		4.44
Hippocampus	R		24	−33	−1		4.98
Cerebellum (Declive)	L		−20	−75	−20	175	3.46
Cerebellum (Tuber of vermis)	R		3	−76	−26	621	4.19
Decreased gray matter density in MDD							
Orbitofrontal cortex	L		−14	46	−18	139	4.08
Orbitofrontal cortex	R	11	20	62	−20	224	3.93
Dorsomedial prefrontal cortex	R	8	15	32	54	149	3.72
Dorsal anterior cingulate cortex	R	24	6	−21	39	153	3.45
Middle occipital gyrus	L		−36	−87	22	292	4.16
Middle occipital gyrus	R	18	38	−88	−8	497	4.71
Cuneus	L	17	−8	−98	10	245	4.40

*Note*: *L* left hemisphere, *R* right hemisphere, *BA* Brodmann’s area; the threshold was set at AlphaSim corrected *p* < 0.05

**Fig. 2:**
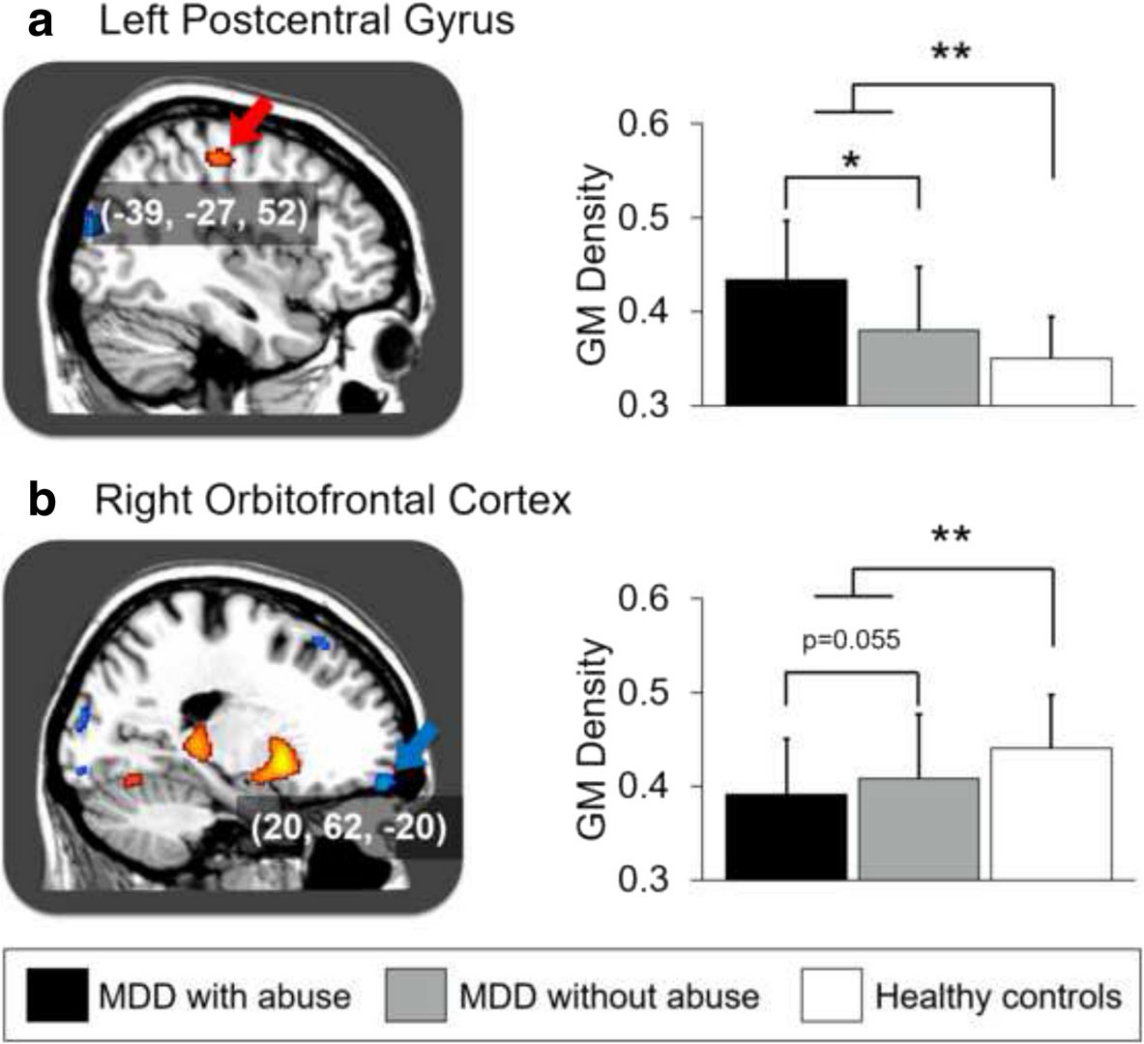
GMD differences between MDD with child abuse, MDD without child abuse, and healthy controls. Clusters colored with red (*blue*) indicate the increased (decreased) GMD in MDD patients, compared to heathy control. Cluster marked with red (*blue*) *arrow* indicate the increased (decreased) GMD in MDD with child abuse, compared to MDD without child abuse. **a** left postcentral gyrus, **b** right orbitofrontal cortex. Abbreviations: GMD, gray matter density; MDD, major depressive disorder. *p < 0.05, **p < 0.001

Within the MDD patient group, the MDD with abuse subgroup showed increased GMD in the left postcentral gyrus, and a tendency of decreased GMD in the right orbitofrontal cortex compared to the MDD without abuse subgroup (Additional file 1 and Fig. 2). There was no significant correlation between GMD and abuse experience scores within MDD patient group.

## Discussion

In this study, we demonstrated that individuals with MDD showed decreased GMD in cortical regions including the OFC, right dorsomedial prefrontal cortex (DMPFC) and posterior cingulate cortex, but showed increased GMD in the subcortical regions including putamen and thalamus compared to healthy controls. In addition, individuals with MDD who also experienced child abuse showed increased GMD in the left postcentral gyrus and a possibility of decreased GMD in the right orbitofrontal cortex.

Our findings suggest that MDD may be associated with structural abnormalities in fronto-striato-thalamic regions, supporting for a cortico-subcortical network dysfunction in the development of MDD [2, 7]. In addition, early-life stressful experiences such as physical or emotional abuse may have an additional impact on brain structure associated with body perception and emotion regulation in MDD patients [22].

Our findings regarding decreased GMD of the bilateral OFC, right DMPFC, and dorsal anterior cingulate cortex are consistent with several prior studies. OFC lesions are known to be associated with a wide range of abnormal affective behaviors including affective instability, irritability, aggression, and depression- or anxiety- related symptoms, which are frequently observed in MDD patients [23, 24]. A postmortem study found significant decreases in cortical thickness of the OFC in MDD patients compared with healthy controls [25]. Several MRI-based studies reported bilateral reduction of OFC volumes in depressed patients [26, 27]. Abnormally increased OFC metabolism or regional cerebral blood flow have been observed in MDD patients [28]. DMPFC lesions also conferred an increased risk of severe depression, compared to lesions outside the prefrontal cortex [29]. A recent meta-analysis of VBM studies showed only minimal GMD changes in the DLPFC, but extensive abnormalities in the DMPFC and adjacent anterior cingulate cortex [30]. Moreover, the DMPFC was identified as a ‘dorsal nexus’ in depression where cortical network, affect regulation, and self-reflections converge in depressed patients [31]. Although still controversial, reduced volume in the dorsal anterior cingulate cortex has been also reported [32–34].

Evidence supporting the possible involvement of the basal ganglia in the pathophysiology of MDD comes primarily from clinical observations. Movement disorders with known basal ganglia pathology such as Parkinson’s disease have associated depressive and other psychiatric symptoms, even before the onset of motor abnormalities [35]. Postmortem studies have also supported an involvement of the basal ganglia in the pathophysiology of MDD [36]. There is a large amount of indirect evidence supporting the presence of extensive dopaminergic, noradrenergic, and serotonergic projections to the basal ganglia, which might constitute the most important neural pathways in the pathophysiology of MDD [37–39]. Although a meta-analysis of brain volume abnormalities in MDD demonstrated moderate volume reductions for the caudate and putamen in individuals with MDD compared to healthy controls, another study reported no significant differences in this region between groups [40–42]. Interestingly, unlike previous results, our study showed increased GMD in the basal ganglia. Basal ganglia are known to be involved in direct and indirect pathway linking various cortical regions to subcortical area. Considering reciprocal connections between frontal and basal ganglia through the cortico-thalamo-striatal circuit, frontal hypoactivity may induce compensatory hyperactivity or increased volume of the basal ganglia. Previous VBM studies in patients with fibromyalgia, which frequently accompanies depressive symptoms, showed increased basal ganglia volume compared to healthy controls [43]. Clinical heterogeneity in patients with MDD may contribute these equivocal findings. Additional large-scale studies focusing on structural and functional changes of the basal ganglia in MDD could clarify equivocal findings in these regions.

Child abuse may have a deleterious long-term impact on the developing brain [8]. Previous studies have revealed that early-life trauma may protract cognitive development, which can be regained after the traumatic situation has ceased; but emotional dysfunction, which is difficult to be recovered spontaneously after early-life trauma has ceased, may increase risk for later psychopathology [44]. MDD patients who have experienced child abuse frequently have problems with regulation of emotions; this is not easily treated with antidepressants and these individuals show a higher prevalence of comorbid borderline personality disorder [45]. In this study, we observed further reduction of GMD in the right orbitofrontal cortex of individuals in the MDD with child abuse subgroup. This might indicate these patients may be more prone to affective instability given the right orbitofrontal cortex is one of the key regions associated emotional dysregulation with in depression [23].

There are several limitations in this study. Comprehensive psychiatric evaluation for personality disorder was not performed even though MDD patients with child abuse may have comorbid personality disorders. Illness history and characteristics of past and current major depressive episodes including duration of MDD episode, age of onset were not also assessed for the MDD patient group. A relatively small number of MDD patients without abuse experiences participated in this study. Medication history and effect of medication was not fully assessed but this effect is not so large since MRI scans were performed at baseline and the very early stage of medication treatment for all patients in this study.

The strength of this study was focusing on the additional variable of childhood abuse in heterogeneous MDD patients. Until now, VBM studies of MDD patients have not paid sufficient attention to accompanying early-life stress or child abuse experiences. Child abuse and early-life stress are important clinical factors in the comprehensive evaluation of MDD patients and for planning prescription of medication and psychotherapeutic approaches. Future neuroimaging studies investigating structural and functional changes in patients with MDD should evaluate histories about their early life. The orbitofrontal cortex may be a region significantly affected by physical or emotional abuse during early life within the MDD patient group.

## Conclusion

Using VBM we found abnormalities associated with child abuse in the right orbitofrontal cortex and left postcentral gyrus regions in a group of MDD patients. This study also replicated previous findings that suggest hypofrontality and limbic/subcortical hyperactivity in patients with MDD. To understand the complex pathogenic mechanism associated with MDD in the brain, further neuroimaging studies with a large number of subjects and sophisticated design should be pursued.

## Additional file

Additional file 1: Comparison of GMD in regions showing significant difference between MDD with abuse subgroup and without abuse subgroup. (DOCX 15 kb)

## Abbreviations

3D-SPGR: Three dimensional spoiled gradient echo
ACC: Anterior cingulate cortex
CSF: Cerebrospinal fluid
DLPFC: Dorsolateral prefrontal cortex
DMPFC: Dorsomedial prefrontal cortex
GM: Gray matter
GMD: Gray matter density
MDD: Major depressive disorder
MNI: The Montreal Neurological Institute
MRI: Magnetic resonance imaging
OFC: Orbitofrontal cortex
SPM: Statistical parametric mapping
TGMV: Total gray matter volume
VBM: Voxel-based morphometry
WM: White matter
