# Supplementary material for: What is the impact of child abuse on gray matter abnormalities in individuals with major depressive disorder: a case control study
Source: BMC Psychiatry. 2016 Nov 14;16:397. doi: 10.1186/s12888-016-1116-y (PMC5109685; doi:10.1186/s12888-016-1116-y)
Supplement: Additional file 1: — Comparison of GMD in regions showing significant difference between MDD with abuse subgroup and without abuse subgroup. (DOCX 15 kb) [file 12888_2016_1116_MOESM1_ESM.docx]

**Supplement Table 1.** Comparison of GMD in regions showing significant difference between MDD with abuse subgroup and without abuse subgroup

| Anatomical Region | Side | BA | MDD  with abuse (N=23) | MDD  without abuse (N=11) | F | p* |  |
| --- | --- | --- | --- | --- | --- | --- | --- |
|  |  |  | Mean ± SD | |  |  |  |
| Increased gray matter density in MDD | | | | | | | |
| Postcentral gyrus | L | 4 | 0.38 ± 0.06 | 0.43 ± 0.07 | 6.512 | 0.017 | |
| Postcentral gyrus | R | 4 | 0.38 ± 0.06 | 0.40 ± 0.06 | 1.213 | 0.281 | |
| Parieto-occipital cortex | L | 31 | 0.60 ± 0.10 | 0.60 ± 0.09 | 0.994 | 0.328 | |
| Parieto-occipital cortex | R | 23 | 0.60 ± 0.09 | 0.61 ± 0.09 | 0.184 | 0.671 | |
| Putamen | L |  | 0.58 ± 0.07 | 0.59 ± 0.08 | <0.001 | 0.983 | |
| Putamen | R |  | 0.55 ± 0.06 | 0.57 ± 0.07 | 0.171 | 0.683 | |
| Thalamus | R |  | 0.47 ± 0.05 | 0.49 ± 0.04 | 0.738 | 0.398 | |
| Hippocampus | L |  |  |  |  |  | |
| Hippocampus | R |  |  |  |  |  | |
| Cerebellum (Declive) | L |  | 0.73 ± 0.09 | 0.73 ± 0.09 | 0.575 | 0.456 | |
| Cerebellum (Tuber of vermis) | R |  | 0.66 ± 0.08 | 0.64 ± 0.07 | 2.300 | 0.142 | |
| Decreased gray matter density in MDD | | | | | | | |
| Orbitofrontal cortex | L |  | 0.34 ± 0.04 | 0.36 ± 0.04 | 1.040 | 0.318 | |
| Orbitofrontal cortex | R | 11 | 0.40 ± 0.06 | 0.39 ± 0.06 | 4.046 | 0.055 | |
| Dorsomedial prefrontal cortex | R | 8 | 0.40 ± 0.07 | 0.42 ± 0.07 | 0.082 | 0.777 | |
| Dorsal anterior cingulate cortex | R | 24 | 0.50 ± 0.06 | 0.52 ± 0.07 | 0.053 | 0.820 | |
| Middle occipital gyrus | L |  | 0.40 ± 0.05 | 0.43 ± 0.07 | 1.769 | 0.196 | |
| Middle occipital gyrus | R | 18 | 0.40 ± 0.07 | 0.39 ± 0.09 | 0.825 | 0.372 | |
| Cuneus | L | 17 | 0.37 ± 0.06 | 0.39 ± 0.06 | 0.050 | 0.824 | |

*Note*: L=left hemisphere; R= right hemisphere; BA=Brodmann’s area; MDD, major depressive disorder patient group

* Analyses of covariance test for continuous variable controlling for age, gender, total gray matter volume, and depressive symptom severity were done.

p-value less than 0.05 was considered significant.
